# Insoles to ease plantar pressure in people with diabetes and peripheral neuropathy: a feasibility randomised controlled trial with an embedded qualitative study

**DOI:** 10.1186/s40814-023-01252-y

**Published:** 2023-02-03

**Authors:** Richard Collings, Jennifer Freeman, Jos M. Latour, Joanne Hosking, Joanne Paton

**Affiliations:** 1 Department of Podiatry, Torbay and South Devon NHS Foundation Trust, Torquay, UK; 2 School of Health Professions, Faculty of Health, University of Plymouth, Plymouth, UK; 3 School of Nursing and Midwifery, Faculty of Health, University of Plymouth, Plymouth, UK; 4 Peninsula Medical School (Faculty of Health), University of Plymouth, Plymouth, UK

**Keywords:** Diabetic foot, Prevention, Offloading, Insole, Feasibility

## Abstract

**Background:**

Therapeutic footwear and insoles are preventative strategies to reduce elevated plantar pressures associated with diabetic foot ulcer risk. An insole intervention appropriate for chairside delivery optimising plantar foot pressure reduction in people with diabetes has been developed.

**Aim:**

To explore the feasibility and acceptability of testing an optimised insole compared with an active control insole to reduce plantar pressures for people with diabetic peripheral neuropathy.

**Methods:**

A double-blinded multi-centre feasibility RCT with an embedded qualitative study. Participants were randomised to either an optimised insole group (intervention) or a standard cushioned insole group (active control). Participants were assessed at baseline, 3, 6, and 12 months with clinical outcomes of foot ulceration and mean peak plantar pressure (MPPP) reduction. An embedded qualitative study involved semi-structured interviews with 12 study participants and three podiatrists to explore their experiences of the intervention and trial procedures. Data were analysed using descriptive statistics (quantitative data) and thematic analysis (qualitative data).

**Results:**

Screened were142 patients from which 61 were recruited; 30 participants were randomised to the intervention group and 31 to the active control group. Forty-two participants completed the study. At 12 months, 69% of the patient-reported questionnaires were returned and 68% of the clinical outcomes were collected. There were 17 incidences of foot ulceration occurring in 7/31 of the active control group and 10/30 in the intervention group. Mean difference in MPPP between the intervention and active control groups for all regions-of-interest combined favoured the intervention. Thematic analysis revealed three themes; accepting the study, behaviour and support during study procedures, and impact from study participation.

**Conclusion:**

The results of the feasibility RCT suggest that the optimised insole holds promise as an intervention, and that a full RCT to evaluate the clinical and cost-effectiveness of this intervention is feasible and warranted for people with diabetic peripheral neuropathy.

**Trial registration:**

International Standard Randomised Controlled Trial Number: ISRCTN16011830. Registered 9th October 2017.

**Supplementary Information:**

The online version contains supplementary material available at 10.1186/s40814-023-01252-y.

## Key messages

What uncertainties existed regarding the feasibility?

Key uncertainties existed relating to key study procedures such as the estimation of recruitment, retention, and adherence rates for the anticipated larger study. Additional uncertainties over the delivery of the house-shoe and insole, the effectiveness of the blinding and the selection of the most appropriate outcome measure provided further endorsement of a feasibility design. These uncertainties are frequently cited in many aspects of diabetic foot trial design.

2) What are the key feasibility findings?

This feasibility study reports that 61 people were recruited, with a 43% recruitment rate. At 12 months, 68.9% completed the study, although there was slight variability by treatment group, and over the follow-up time points. At 12 months, 69% of the patient-reported questionnaires were returned and 68% of the clinical outcomes were collected. There were 17 incidences of foot ulceration occurring in 7/31 of the active control group and 10/30 in the intervention group. Mean difference in plantar pressure between the intervention and active control groups for all regions-of-interest combined favoured the intervention, with increases from 87 kPa at post-randomisation to 255 kPa at 12 months. Thematic analysis revealed three themes: accepting the study, behaviour and support during study procedures, and impact from study participation.

3) What are the implications of the feasibility findings for the design of the main study?

The results and findings from the feasibility study demonstrate that the optimised insole intervention holds promise. However, refinements to some of the study procedures and recruitment of participants that better represents the general population with diabetes at risk of DFU are recommended. Based on these recommendations, the next step would be to design and implement a powered RCT to evaluate the clinical and cost-effectiveness of the optimised insole intervention.

## Background

Diabetic foot ulceration (DFU) is a multi-factorial complication of diabetes. At any one time, the National Diabetes Footcare Audit reports that approximately 64,000 people in the UK have a diabetic foot ulcer [1]. The Global Burden of Disease study ranks diabetes mellitus-related lower extremity complications as 10th on a scale of leading causes of global years lived with disability in 2015 [2, 3]. In 2014–2015, estimates of costs attributed directly to DFU and lower limb amputation in the National Health Service (NHS) in England was around £1 billion [4].

The most effective way to reduce DFU is through prevention. Prevention of first diabetic foot ulcer or recurrence of DFU is best achieved by a combination of strategies [5]. One preventative strategy is through therapeutic footwear and insoles that have pressure-relieving effects [6]. However, despite high-quality systematic review evidence that supports the use of therapeutic footwear and insoles to prevent the risk of DFU [7–9], there is substantial diversity and variation in efficacy [10], design, and with frequent delays in delivery to patients. This uncertainty can make the use of therapeutic footwear and insoles ill-timed, with difficulties in evaluation and in predicting effectiveness for people at risk of DFU.

Using the Medical Research Council’s (MRC) framework for developing and evaluating complex interventions [11], which has been recently updated [12], an optimised insole intervention was designed to address the uncertainties associated with therapeutic footwear and insoles in reducing DFU risk. The optimised insole intervention uses in-shoe plantar analysis to gather real-time pressure mapping to inform its design to reduce elevated plantar pressures. The insoles are appropriate for chairside delivery and issued to patients within a single outpatient visit, reducing issuing delays. Consequently, the optimised insole intervention provides an objective and standardised approach using quantitative evaluation to design, modify, and monitor the insole’s performance in reducing peak plantar pressures. Having developed the optimised insole intervention, the MRC framework for developing and evaluating complex interventions suggested a feasibility or pilot stage [11]. The intention was to address critical uncertainties in trial design and research questions before moving to a future powered/definitive RCT to determine clinical and cost-effectiveness.

Uncertainties existed over the delivery of the optimised insole intervention, the effectiveness of the blinding strategies, and the selection of the most appropriate outcome measure, which are frequently cited in diabetic foot trials [13, 14]. Therefore, a feasibility RCT (fRCT) with an embedded qualitative study was chosen [15]. Undertaking a fRCT addresses acceptability, compliance, delivery of the intervention, recruitment, and retention of participants, and inadequate effect sizes before full-scale evaluation [16]. Furthermore, exploring study participants’ and clinicians’ experiences can provide valuable insight into the procedural, methodological and clinical issues of the study and are crucial to refining the design of a research study [17]. Therefore, this fRCT aimed to test an insole prescription and fabrication intervention appropriate for chairside delivery to reduce plantar foot pressures and consequent foot ulceration risk in people with diabetic peripheral neuropathy.

Specifically, the objectives were to

1. Assess the feasibility and acceptability of the trial procedures comparing the optimised insole plus usual care with an active control insole plus usual care for people with diabetic peripheral neuropathy (DPN);
2. Assess the appropriateness and performance of outcome measures to select the most appropriate primary and secondary outcome measures;
3. Assess for a signal of efficacy and inform the sample size calculation of a future RCT;
4. Explore the experiences of participants receiving the interventions and the podiatrist’s experiences of delivering the intervention.

## Methods

A participant and assessor-blinded, randomised, multi-centre, parallel-group feasibility trial with an embedded qualitative study was conducted. The methodology of the fRCT is described briefly below, with more detail in the published protocol [18].

### Settings

The fRCT was conducted between November 2017 and January 2019 at three study sites located in the South-West of England. Historically, diabetes‐related lower limb amputation incidence has been very high across most of the South‐West of England [19]. This variation is associated with differences in demographics, where an ageing population and higher proportions of people with diabetes and its coexisting complications are present compared to other areas of England [20].

### Participants

Participants were aged 18 years and above with a confirmed diagnosis of type 1 or type 2 diabetes and the presence of sensory DPN, defined as the insensitivity of a 5.07/10 g monofilament at one of three sites on each foot [21, 22]. Additionally, they were required to have a clinical need for insoles, needing an offloading device due to a recently healed or healing ulcer site on the weight-bearing plantar surface of the foot, and/or pre-ulcerative callus formation.

Participants were excluded if they had a non-healing foot ulcer at another site on the plantar aspect of the foot, gross foot deformity, e.g., Charcot foot or fixed rearfoot deformity, or had undergone a major amputation. Additionally, those with non-re-constructible peripheral vascular disease, lacking capacity or unwilling to give consent, unable to walk 5 m or stand on either leg independently for 10 s and were already wearing existing insoles or unwilling to wear therapeutic footwear were excluded.

### Recruitment

The podiatry clinical team identified potential participants at each participating site during a routine appointment within the multi-disciplinary diabetic foot clinic or podiatry community clinic. Only those appearing to meet the eligibility criteria for inclusion in the study were approached. Potential participants were given a brief verbal explanation of the study by the podiatrist and provided with a participant information sheet.

### Screening and consent

Following identification of potential participants, screening was undertaken by a research nurse who contacted the individual after a minimum of 24 h of receiving the participant information sheet to discuss the study in more detail and provide the opportunity for them to ask further questions. If eligibility criteria were met, a podiatry appointment was arranged, where final confirmation of eligibility was undertaken.

Following written informed consent, baseline assessment consisted of collecting demographic data, self-reported activity and self-care questionnaire, photograph of foot status and pressure analysis with F-scan (including gait style definition). The F-scan (Tekscan, Boston, MA) is an in-shoe measurement system based on resistive sensor technology that is capable of reliable and repeatable data collection [23]. The data aimed to capture both objective and subjective changes in foot health, health behaviour, and activity modification as well as enabling scrutiny of any confounding factors in post-trial analyses.

### Randomisation

At the same visit, immediately following baseline assessment by the podiatrist, eligible participants were randomied in a 1:1 allocation to one of two groups: one group received the optimised insole designed to reduce peak plantar pressure in addition to usual care (intervention); while the other group received a cushioned inlay insole in addition to usual care (active control). The randomised allocations were generated by computer, with input from an independent statistician. Participants’ details were entered into the randomisation website by the podiatrist after the baseline assessment. Immediately following randomisation, the podiatrist received an email indicating the participant's group allocation and subsequently provided them with either the optimised insole or active control insole.

### Sample size

As a fRCT, no formal power calculation was undertaken (Eldridge et al. 2016b). However, the overall target recruitment was 76 participants (38 per group) at an anticipated rate of two per month per centre. A confidence interval approach was used to establish completion rates. Based on an estimated completion rate of 75%, at least 75 patients were required. This was based on obtaining a 95% confidence interval (CI) for a single proportion with a specified lower bound of the CI of 0.70 and a marginal error of 0.05.

### Procedures

Participants were asked to put on standard stocking socks (20 denier) and were fitted with a standard house shoe (Pulman, M. J. Markell Shoe Co. P. O. Box 246 Main Station, Yonkers, NY 10702–0246, USA). The F-scan in-shoe pressure analysis system’s sensors were connected to a computer via a cuff unit and a 9.14-m-long cable. Following acclimatisation and calibration of the equipment, data was collected at a sampling rate of 50 Hz for 4 s.

Participants were asked to undertake two test walks immediately after calibration between a pre-marked 5-m walkway at their usual walking speed [24, 25]. A minimum of three mid-footsteps for peak pressure data collection were analysed, with the first and last steps discarded for acceleration and deceleration effects. Three mid-foot steps are recommended for peak plantar measurement based on having excellent reliability (ICC ≥ 0.90) [26].

A maximum of three regions of interest (ROI) across both feet were identified for each participant, where ROI was a recently healed ulcer site(s) or callus/corn formation, and/or where the mean peak plantar pressure of the ROI was greater than 350 kPa. Also, identifying the type of gait style (Propulsing gait, Stomping gait, Variable gait) by analysing the recorded F-scan pressure–time curve and force–time curve occurred [27].

### Intervention

Two different insoles were evaluated for feasibility and acceptability in this trial: instant optimised insole (intervention) and cushioned inlay insole (active control). Both insoles were custom-made to foot size and constructed using materials commonly used to manufacture insoles for people with diabetes. Each insole was fitted into a Pulman house shoe, measured to fit the participant’s foot. Also, both insoles had an activated data logger (Orthotimer, Algeos, Liverpool, UK) embedded into the insole to measure adherence to wearing the insole.

Every effort was made throughout the study to ensure participants were blinded to their group allocation. The optimised insole and active control insole received identical top covers to minimise discovery of group allocation. Insoles were fitted into the participant’s house shoe by the podiatrist to minimise handling and inspection of the insole by the participant. Peak pressure data was not revealed to the participant to minimise the potential for unblinding. The podiatrists were unable to be blinded to the intervention as they manufactured and provided the optimised and active control insole.

### Intervention group (optimised insole)

The intervention group received instant customised insoles designed and optimised using the F-scan in-shoe pressure analysis system and a Pulman house shoe. The optimised insole consisted of a pre-constructed base to conform to the foot's contours (Slimflex Full length Medium Density (Shore A50), Algeos, UK). The insole design and modification(s) were informed by the novel treatment algorithm based on walking gait style. Regions of interest were identified to accommodate for prominent areas, previously ulcerated areas or areas of high mean peak plantar pressure. These areas were targeted with modifications designed to offload pressures and a 3 mm Poron ® 4000 top-cover. These modifications were used to reduce mean peak plantar pressure (MPPP) values in conjunction with real-time pressure data from the F-scan system in the specific ROI.

### Active control group 

The active control group received a 3-mm flatbed insole of Poron® 4000 with a 3-mm medium-density heel lift and a Pulman house shoe. Previous research demonstrated that a 3-mm flat medium density polyurethane (Shore A hardness 55 ± 3) insole reduced peak pressure under 1st MTPJ by 35 kPa compared to shoe-only condition in healthy participants [28].

After the initial baseline visit, all study participants were invited to attend three further assessment appointments at 3, 6, and 12 months post-randomisation. These visits were in addition to the usual care monthly podiatry appointments.

### Outcome measures

Objective one was to assess the feasibility and acceptability of the trial procedures as per the CONSORT extension for Pilot and Feasibility Studies [29, 30]. In particular:

- The numbers of participants screened, eligible, randomised, and withdrawn from the study by study site and group allocation;
- The completeness of the data sets and follow-up rates, and the number of missing observations for each characteristic;
- The effectiveness of the participant and assessor blinding using the Blinding Index (BI) [31].

Objective two was to determine the most appropriate primary and secondary outcome measures to inform the anticipated future RCT by measuring:

- Incidence of plantar foot ulceration, through photographs of each foot and self-report by participants and podiatrists;
- The proportion of completed questionnaires items in the Nottingham Assessment of Functional Footcare questionnaire [32]; and the International Physical Activity questionnaire [33];
- Adherence to wearing the insole, as assessed by a temperature sensor (Orthotimer, Rollerwerk Medical Engineering, Balingen, Germany) integrated into the optimised and active control insoles;
- Assessment of trial safety by adverse event data.

Objective three was to estimate various parameters needed to calculate an indicative sample size for the anticipated RCT:

- Signal of efficacy, by evaluating the MPPP effect estimates as assessed by considering appropriate confidence intervals of the mean, standard deviation of MPPP, and the correlation between baseline and follow-up MPPP;

Objective four was to explore the experiences of participants receiving the interventions and podiatrists’ experiences of delivering the intervention.

### Embedded qualitative study

Participants’ experiences of receiving either the customised, optimised insoles and Pulman-house shoe, or active control insole and Pulman house shoe, and the podiatrists’ experiences of delivering the intervention were explored using semi-structured interviews [34]. A purposive sampling approach was used to achieve maximum variation in previous foot ulcer history, gender, and age, to explore the experiences of 12 patient participants (4 from each study site; 2 from the intervention group, and 2 from the active control group) and three podiatrists (one from each study site) delivering the intervention. Participants were interviewed 4 months after randomisation and podiatrists 6 months after the study site commenced recruitment.

### Data analyses

Statistical analyses were undertaken using Statistical Package for the Social Sciences (SPSS) version 25 (IBM Corporation, Released 2016), supplemented where required by Stata SE Version 14.0 and R (www.r-project.org). Continuous variables were summarised as mean (standard deviation) and median (interquartile range) with 95% CI, while categorical variables were summarised as frequency and percentage. As an indication of a signal of efficacy [35], the between-group differences of the change in MPPP from baseline to each follow-up time-point were calculated. Both the unadjusted and adjusted for baseline plantar pressure (to account for possible regression to the mean) data were calculated using analysis of covariance. Thematic analysis based on the six steps described by Braun and Clarke was utilised to investigate the transcribed interview data [36]. Briefly, this entailed transcription and independent coding of the data using qualitative data analysis software NVivo (v12.0). This was followed by creating thematic maps to visualise links and relationships between codes and sub-themes and discarding codes that were too diverse or not supported by sufficient data. Finally, themes were identified and illustrative narratives prepared.

## Results

**Fig. 1:**
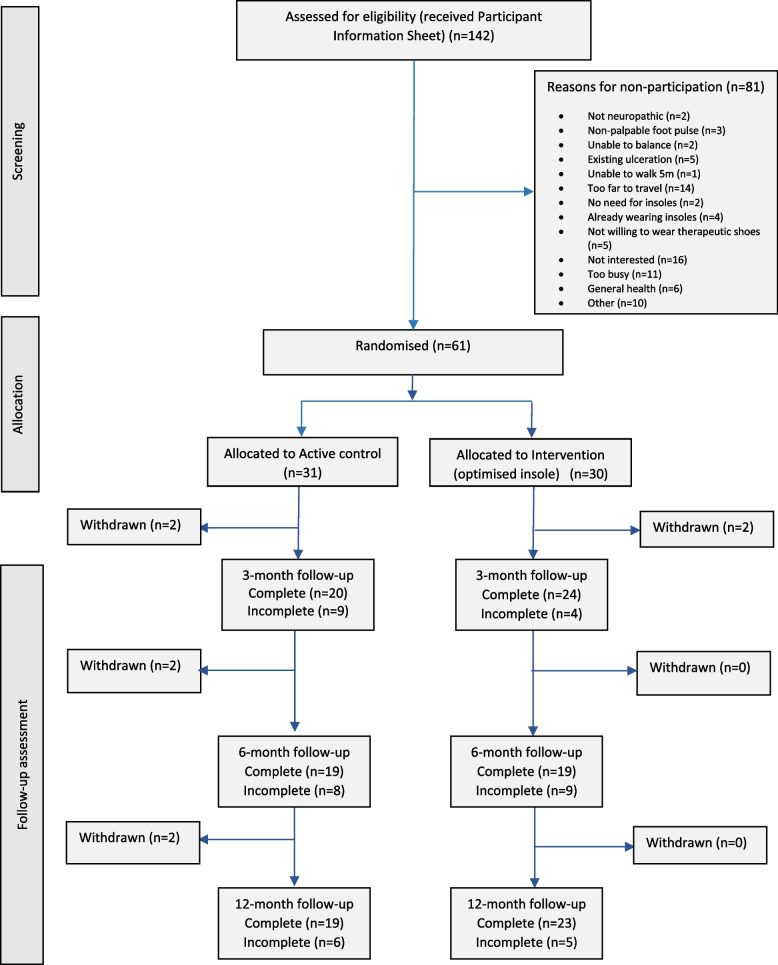
CONSORT patient flow through the INSTEP trial

In total, between November 2017 and December 2018, 142 potentially eligible patients were screened and received a patient information sheet (Fig. 1) After screening, 57% (*n* = 81/142) were not considered for further study participation. Forty-three percent (*n* = 61/142) of the target population were recruited and randomised to the active control group (*n* = 30) or intervention group (*n* = 31). The study participant’s characteristics are presented in Table 1.

**Table 1:** Baseline demographics by treatment group allocation

		Active control *n* = 31	Intervention (optimized insole) *n* = 30
Age (years)	Mean (SD)	67.9 (12.2)	70.2 (10.2)
	Median (IQR)	70.0 (60.0–73.0)	71.5 (67.8–74.8)
Gender, *n* (%)	Female	5 (16.1%)	3 (10.0%)
	Male	26 (83.9%)	27 (90.0%)
Height (cm)	Mean (SD)	177.2 (11.0)	176.1 (9.1)
	Median (IQR)	178.0 (171.5–183.5)	177.0 (171.0–183.0)
Weight (kg)	Mean (SD)	95.0 (14.1)	94.4 (18.6)
	Median (IQR)	94.0 (85.0–107.0)	92.0 (79.6–111.3)
BMI (kg/m^2^)	Mean (SD)	30.4 (4.6)	30.4 (5.5)
	Median (IQR)	29.8 (37.7–32.2)	29.5 (20.3–35.8)
Ethnicity, *n* (%)	White	31 (100%)	30 (100%)
Smoker, *n* (%)	Yes	3 (9.7%)	2 (6.7%)
	No	28 (90.3%)	27 (90.0%)
	Missing	0 (0)	1 (3.3%)
Diabetes type, *n* (%)	Type 1	7 (22.6%)	2 (6.7%)
	Type 2	24 (77.4%)	28 (93.3%)
Duration of diabetes (years)	Mean (SD)	21.3 (9.7)	19.7 (14.9)
	Median (IQR)	20.0 (14.5–27.5)	17.0 (6.0–28.5)
Previous foot ulceration *n* (%)		16 (51.6%)	15 (50.0%)
Current foot ulceration *n* (%)		0 (0%)	0 (0%)

%’s are expressed as the proportion of group allocation

Follow up closed on 31st January 2020. There was variability in the number of participants completing the study follow-up visits at 3, 6, and 12 months post-baseline (3 months 72.1%, 6 months 62.3%, 12 months 68.9%). The proportion of participant completing study follow-up assessment differed depending on group allocation (active control group—3 months 64.5%, 6 and 12 months 61.3%; intervention group—3 months 80%, 6 months 63.3%, 12 months 76.6%).

At 12 months post-randomisation, 33.1% (*n* = 19/61) of participants were lost to follow-up with 13.1% (*n* = 8/61) specifying reasons. These were: moving out of the area (*n* = 3), death (*n* = 1), ongoing foot ulceration (*n* = 1), study too burdensome (*n* = 1), ill-health (*n* = 1), no reason (*n* = 1).

There was minimal variation in the proportion of completed data sets between the intervention and active control groups. There was no missing data for MPPP and photographs for those who attended the follow-up sessions. At 3 months follow-up, 72.1% (*n* = 44/61) of MPPP measurements and photographs assessing foot ulcer status were collected. At 6 months, 60.7% (*n* = 37/61) and at 12 months follow-up, 67.2% (*n* = 41/61) of MPPP data sets were collected. At 6 months 62.2% (*n* = 38/61) and at 12 months follow-up 68.9% of photograph data sets (*n* = 42/61) were obtained.

The Bang Blinding Index assessed participant blinding to the treatment allocation across both groups at the 3, 6, and 12 months follow-up period. The BI index ranged from − 0.26 to 0.2 for the active control group and − 0.476 to 0 for the intervention group indicating that an excellent level of blinding of participants to the intervention was maintained.

### Outcome measures

There were 17 incidences of foot ulceration during the 12 months of the study, occurring in 22.5% (*n* = 7/31) of the active control group participants and 33.3% (*n* = 10/30) of the intervention group. Foot ulceration was defined as a break in the skin epidermis, and all occurrences were determined by self-report of adverse events. Of the seven incidences of ulceration in the active control group, six were attributable to trauma and one to a pressure related causation. In the intervention group, seven incidences were trauma-related and three to a pressure-related causation.

The protocol directed that up to three ROI for each participant could be selected for the podiatrists’ analysis. As an indication of a signal of efficacy, the between-group differences of the change in MPPP from baseline to each follow-up time-point were calculated for ROI-1, ROI-2, and the mean of all ROI’s (Table 2). ROI-3 was not calculated due to the low numbers within this sub-sample. Assessing ROI-1, ROI-2, and ROI-combined ensured that any effect from the intervention on the other regions-of-interest was also evaluated. Both the unadjusted and adjusted baseline plantar pressure (to account for possible regression to the mean) data are presented using analysis of covariance.

**Table 2:** Change from baseline in plantar pressures by treatment group allocation for region of interest-1, region of interest-2, and all regions combined at each time point

	Region of interest -1				Region of interest-2				All regions of interest combined	
Time point	Active insole Mean MPPP kPa (sd)	Optimised insole Mean MPPP kPa (sd)	Mean MPPP difference unadjusted for baseline kPa(CI*)	Mean MPPP difference adjusted for baseline kPa(CI*)	Active insole Mean MPPP kPa (sd)	Optimised insole Mean MPPP kPa (sd)	Mean MPPP difference unadjusted for baseline kPA (CI*)	Mean MPPP difference adjusted for baseline kPA (CI*)	Mean MPPP difference unadjusted for baseline kPA (CI*)	Mean MPPP difference adjusted for baseline kPA (CI*)
Baseline	564.0 (223.0)	583.3 (220.9)	n/a	n/a	499.4 (240.4)	583.3 (220.9)	n/a	n/a	n/a	n/a
Immediately post randomisation	447.4 (181.9)	370.2 (162.1)	96.3 (CI 29.4, 163.3)	89.2 (CI − 36.7, 141.7)	452.7 (252.2)	360.9 (157.3)	161.5 (CI 37.1, 285.9)	136.7 (CI 26.8, 246.6)	100.5 (CI 36.6, 164.4)	87.9 (CI 40.1, 135.7)
3-month follow-up	546.1 (229.6)	495.9 (244.4)	123.7 (CI − 286.4, 39.0)	77.3 (CI − 61.5, 216.2)	648.6 (378.9)	495.9 (244.4)	203.1 (CI − 13.6, 419.9)	187.3 (CI − 28.8, 403.5)	155.4 (CI 11.4, 299.5)	122.2 (CI − 5.0, 249.5)
6-month follow-up	639.8 (332.3)	625.3 (353.8)	51.4 (CI − 140.3, 243.1)	44.5 (CI − 148.5, 237.5)	717.0 (476.6)	525.3 (253.8)	247.7 (CI 3.5, 491.8)	283.9 (CI 36.0, 531.8)	124.2 (CI − 53.0, 301.3)	112.0 (CI − 63.4, 287.4)
12-month follow-up	854.7 (538.9)	596.2 (437.6)	238.9 (CI − 32.1, 509.9)	239.4 (CI − 35.4, 514.2)	797.1 (592.7)	596.2 (437.6)	252.4 (CI − 61.0, 565.8)	283.6 (CI − 55.9, 623.1)	257.9 (CI 15.9, 500.0)	255.5 (CI 10.1, 501.0)

*CI* confidence intervals expressed as 95%, *kPa*  kilopascal, *sd* standard deviation

The Nottingham Assessment of Functional Footcare Questionnaire [32] was used to assess the participants’ engagement with their foot care, and the International Physical Activity Questionnaire [33] was used for participants to self-evaluate their activity levels. The proportion of both questionnaires returned at baseline was 98.3% (60/61). Returns were consistent across the follow-up time points and across groups (3-month, 72.1% (*n* = 44/61), 6-month, 62.3% (*n* = 38/61), 12-month, 68.9% (*n* = 42/61) with more than 80% of questions completed at each time point.

Data logger uploads were completed for forty-four participants to record insole wear time with slight variation for wear time across treatment groups. 45.5% (*n* = 20) of participants wore the insoles for less than 4 h each day, 38.6% (*n* = 17) wore insoles for 4 to 8 h, and 15.9% (*n* = 7) for more than 8 h per day. No thresholds for wear time to indicate adherence had been set, although it was considered that high adherence was greater than 8 h of daylight [37].

There were 26 Adverse Events (AE) involving 17 participants and 6 SAE’s (SAE) involving 3 participants for the duration of the study. Of the AE’s reported, 34.6% (*n* = 9/26) were in the active control group, and 65.4% (*n* = 17/26) were in the intervention group. Ten were considered attributable to the trial intervention (6 in the intervention group) and 16 not attributable to the intervention (9 in the intervention group). Of the 17 incidences of ulceration, 16 were considered not attributable to the intervention. All SAEs were unrelated to the trial intervention.

### Qualitative results

Thematic analysis of the interviews revealed three themes: (1) accepting the study; (2) behaviour and support during study procedures; (3) impact from study participation. A summary of the narratives of the patient participants (Additional file 1) and podiatrists (Additional file 2) are presented.

The theme ‘Accepting the study’ indicated that the study procedures were acceptable to study participants and podiatrists. Sub-themes identified specific elements of the study procedures that were acceptable and identified areas that required improvement for a potential future larger RCT. Involvement in the study and the randomisation process was acceptable to most patient-participants. However, some participants deemed the patient information sheet as too lengthy and easy to forget and some participants raised issues about the lack of applicability of some of the categories in the patient-reported outcome questionnaires, although completing them was acceptable. Most patient-participants found the assessment process for receiving the footwear and insoles acceptable, apart from difficulty performing the calibration task. Podiatrists highlighted the provision of the house-shoe and production of the insole as straightforward, although safety concerns over tripping were raised over the house-shoe. Improvements to address the concerns of the podiatrists relating to the technical constraints imposed by the NHS information technology systems, where NHS systems in some Trusts prevented the software from being downloaded.

The theme ‘Behaviour and support during the study procedures’ revealed differences in the insights into the patient participants’ experiences and the podiatrists’ conduct during the study period. Sub-themes identified diverse participant behaviours regarding self-foot-care activities, which can influence foot ulceration risk. Different motivations influenced the decision to participate in the study. Some patient participants had altruistic motivations, while others hoped they would receive improved treatment for their foot care. Some participants highlighted the supportive contribution of family members and how this influenced actions relating to using footwear and insoles and managing their diabetes condition during the study. The existing therapeutic relationship of support provided by podiatrists when recruiting was emphasised as an essential factor in participant recruitment and enabled a positive experience for patient-participants. Podiatrists were equally conscious of the positive interface with the participants during the study. They related the satisfaction of having the time to explain the study processes to participants. Podiatrists also found benefit from the support of the wider research team which supplemented the training programs delivery of the study procedures. However, some modifications would benefit the anticipated RCT.

The theme ‘Impact of study participation’ revealed different aspects of the study that impact the patient participants and podiatrists. Sub-themes revealed the impact of the clinic location and the struggles that some participants with diabetes had in accessing the location. This is an important consideration for the anticipated RCT. Patient participants highlighted the impact of living with diabetes and frequently motivated by a fear of foot ulceration and amputation. Overall, participants enjoyed taking part in the study, finding it interesting and not burdensome, which is important to recruitment and retention rates. There was a common desire to receive feedback about the study outcomes, although the preferred mode of delivery for the dissemination varied amongst the patient-participants. A sub-theme relating to overall learning was highlighted by the podiatrists, who reported impacts of positivity and enjoyment from their contribution to the study. They highlighted the impact of the changes in their clinical practice due to their involvement in the study and recognised the influence dissemination of the study results could have on their colleagues.

## Discussion

The purpose of this fRCT was to lay the foundation for a future definitive RCT to examine the clinical and cost-effectiveness of an optimised insole for people with diabetic peripheral neuropathy at risk of foot ulceration. Valuable insights and lessons have been learned, with recommendations developed. The recommendations are designed to improve the operationalisation of a future definitive trial.

### Study procedures

The recommendations from the study procedures focussed on lessons from participant recruitment, retention, and completion rates; suitability and feasibility of eligibility criteria; randomisation; blinding and adherence, all of which were pre-defined study objectives. Recruitment and retention of participants were lower than anticipated. Recruitment to multi-centre RCTs can be particularly challenging, with a survey of UK clinical trials reporting participant recruitment as the most common inefficiency [38]. Therefore, it will be important to utilise those strategies that were successful in augmenting recruitment, which included targeting potential participants through personal invites by the podiatry team and active engagement and support by podiatry team members. This concept is supported by thematic data analysis from clinicians’ focus group discussions in a UK-based multi-site RCT [39]. The authors emphasised the necessary engagement to ensure that clinical staff were both educated and motivated to help with the process of identifying and screening potential prospective participants for the trial.

Reducing the recruitment target rate to 1–2 participants per month per site would seem realistic for a larger trial to allow for operational delays and seasonal variations experienced in the fRCT. To reduce attrition, ensuring a positive relationship between the participant and podiatrist to enable a personalised approach is recommended. Strategies will include the use of reminder letters for appointments and a greater degree of flexibility in the clinical location to deliver the research assessments in community-based health care establishments and more available time slots for participants to attend. Eligibility criteria were pragmatic and easy to apply and are relevant for a future RCT, with the stratified randomisation generating homogenous intervention groups. However, strategies for recruiting participants of mixed ethnicities and gender that better represents the general population with diabetes at risk of DFU are recommended. For example, providing patient information leaflets in a range of languages, translating technical phrases and involving a culturally competent person would be recommended.

Using an active control insole enabling identical insole top-covers for each intervention group proved effective for achieving optimal participant and assessor blinding and standardised delivery of the active control across sites [40]. However, the inclusion of a third “usual care only” arm in a future RCT deserves consideration to evaluate the optimised insole against usual care. This, however, has the disadvantage of significantly increasing study complexity, sample size, costs, and impact on the blinding. The use of the sensor to objectively measure adherence to wearing the insole was successful, where proportions of wear time were comparable with other studies that used similar sensor-based technology [37, 41]. This information is particularly relevant in people at risk of DFU, where review studies have described the under-adherence of wearing footwear and insoles prescribed for offloading [8, 9, 42]. The technological advances and wider availability in diabetic foot Apps also provide another option for adherence data collection. We suggest a more comprehensive evaluation of variations to the insole wear time relative to the time spent wearing other footwear throughout the study duration should be attempted.

### Study outcomes

Data on participant demographics, clinical characteristics, and a range of potential primary and secondary outcomes were collected to inform a future definitive trial. The completion and performance rates of the measures were excellent, clearly meeting our pre-defined progression criteria for a definitive trial [43].

One aim of this fRCT was to select the primary outcome to be used in a future RCT. In the absence of recommended core outcome measures for studies for DFU prevention, the primary outcome measures considered were MPPP and incidence of DFU. The completeness and performance were excellent and met the criteria set for progressing to a definitive trial. Both measures provided information for analysis, DFU incidence was collected from the reporting of safety data, although proved problematic throughout the study. Photographs, whilst a feasible method of capturing foot status and enabling the blinding of clinicians, were restricted to fixed time points and not sensitive to DFU events. Whereas MPPP was easily obtained at follow-up. but is considered a surrogate measure of DFU, widely used in studies to evaluate the effectiveness of footwear and insole interventions in people with diabetes mellitus. One systematic review identified that 72% of studies used kinetic outcomes, such as MPPP, as the primary outcome measure when evaluating therapeutic footwear and insole intervention for people with DPN at risk of DFU [44]. Yet, whilst MPPP enables inference of the signal of efficacy for the intervention group compared to the active control group, the normal or acceptable values for MPPP have not been validated. Resultantly, the conclusion drawn based on this fRCT is that the primary outcome measure for a future trial would be DFU occurrence However, MPPP and the number of diabetic foot ulcer-free days would be valuable secondary outcomes measures, which are particularly helpful if they lend support to the primary endpoint [45].

Within this study, whilst there was a greater incidence of foot ulceration in the intervention group, the underlying cause of the ulcer was unusual and could not have been prevented by the use of insoles. The number of plantar neuropathic foot ulcer occurrences in occurring at areas of high pressure occurring in either group over the duration of the study were very few. Consequently, collection of baseline variables will be crucial to record so that confounders which may cause DFU, are incorporated within the statistical analysis, thereby enhancing interpretability of the results. Similarly, the gathering data on the location and causal mechanism (e.g. pressure, trauma, vascular) and plotting the route causation of any DFU will be equally important to identify its viability to plantar pressure relief.

We had originally proposed that MPPP would be the primary outcome of interest and, as such, we calculated sample sizes for a definitive trial for a range of scenarios with varying assumptions. In the absence of clinical information regarding what constitutes a clinically important difference in MPPP reduction, we assumed for example that a difference of 160 kPa (30% MPPP reduction from baseline of all ROI’s) would be considered of minimal clinical importance, translating to a standardised effect size of 0.4, usually considered small-to-moderate. Using the correlation coefficient of 0.55 to improve the precision of the estimate and an allowance of 30% for drop-out, it is estimated that the multi-centre trial would require 265 participants in total to provide 90% power at the 5% (two-sided) significance level. Should the primary outcome be a difference in the proportion of participants who experience foot ulceration, it is acknowledged that this may require a larger sample size, being dependent on the anticipated proportion of participants who experience foot ulceration, which is likely to vary according to previous history of ulceration. As an example, a total sample size of 824 would achieve 90% power to detect a difference between group proportions of 0.1, assuming the proportion in the intervention group is 0.2 under the null hypothesis and 0.3 under the alternative hypothesis, with the proportion in the active control group being 0.2.

### The intervention

Overall the concept and delivery of the optimised insole appeared acceptable to participants and podiatrists, although recommendations to fine-tune these aspects are necessary. Ideally the follow-up period should extend to 24 months to enable more comprehensive assessment of the effectiveness of the intervention insole compared to the active control insole. Consideration of an intention to treat analysis may be beneficial to overcome problems of missing data and withdrawals over the 24-month follow-up period. Additionally, this will enable evaluation of the insole durability and long-term patient adherence to insoles over time. The delivery of the insole intervention would be improved by addressing some of the operational constraints (appointment times and clinical locations) highlighted in the fRCT. However, this will have implications on the study setup. In particular, incorporating administration time for the podiatrists and providing prospective IT support is recommended. One systematic review (2018) suggested that the clinician time burden could be partly responsible for the lack of engagement [46]. Speculatively, there is a gap in knowledge about the best ways to engage with podiatrists in delivering research studies. The training package for delivering the insole intervention and study procedures was demonstrated to be generally fit for purpose. It could be used in the future study, with some minor modifications. Active learning, the approach used for training in this study, included clinical simulations, alongside practice and feedback has been identified as effective educational technique for health professionals [47]. The importance of the positive therapeutic relationships between participants and podiatrists during the study necessitates more formal recognition of the need to support participants and their family and friends in conjunction with developing a structured support network for podiatrists during the study [48].

## Limitations

While the signal of efficacy suggests that the optimised insole shows promise in reducing MPPP, there are uncertainties over the durability of the insoles and their ability to maintain this reduction for longer than 6 months effectively. Replacement of worn insoles would have cost implications for a future study that has not been taken into account.

All study sites were in the South West of England, where the ethnic and cultural diversity of both the study participants and podiatrists is lower than the national average and therefore not representative of the broader population. In the embedded qualitative study, only one female agreed to be interviewed, further limiting the findings' transferability. In addition, the interviews were undertaken after participants had completed the 3-month follow-up appointment while they were still actively engaged in the trial. Therefore, the participant views were only representative of the early part of the study and did not include the perspectives of those who dropped out of the study.

## Conclusions

The results of our fRCT suggest that the optimised insole holds promise as an intervention in preventing DFU in people with diabetic peripheral neuropathy. Subsequently, a fully powered RCT to evaluate the clinical and cost-effectiveness of this intervention is feasible and warranted. Study procedures were generally acceptable with recommendations to improve the design of a full RCT. Completion of the study outcome sets were successful in the fRCT, with ulceration occurrence as the most important primary outcome measure for any future RCT. However, uncertainty exists regarding the length of time that the optimised insole may be effective in reducing MPPP and this should be investigated further in the main RCT.

## Supplementary Information

**Additional file 1. **Identified narratives of themes and sub-themes from patient participant interviews.

**Additional file 2. **Identified narratives of themes and sub-themes from podiatrist interviews.

## Data Availability

The datasets used and/or analysed during the current study are available from the corresponding author on reasonable request.

## Abbreviations

DFU: Diabetic foot ulceration
DPN: Diabetic peripheral neuropathy
fRCT: Feasibility randomised controlled trial
MPPP: Mean peak plantar pressure
RCT: Randomised controlled trial
