# Supplementary material for: Insoles to ease plantar pressure in people with diabetes and peripheral neuropathy: a feasibility randomised controlled trial with an embedded qualitative study
Source: Pilot Feasibility Stud. 2023 Feb 3;9:20. doi: 10.1186/s40814-023-01252-y (PMC9896776; doi:10.1186/s40814-023-01252-y)
Supplement: Supplementary file 1 — Additional file 1. Identified narratives of themes and sub-themes from patient participant interviews. [file 40814_2023_1252_MOESM1_ESM.docx]

Additional File 1 Identified narratives of themes and sub-themes from patient participant interviews

| **Theme** | **Subtheme** | **Patient participant narratives** |
| --- | --- | --- |
| **Accepting the study methods** | Participant recruitment | “Well, because I was asked. It was someone from the clinic. They checked my feet and asked me if I wanted to take part. So I did.” (0203) |
|  | Participant Information | “Well, I understood all the information sheets. Yes and all it entailed, yes I found it quite clear and precise and there was no unexpected things as the trial went on, I didn’t say, oh I never knew that there was nothing of that.” (0103)  “Too long ago to remember! (laughed loudly!) I remember it was long and very detailed.” (0315) |
|  | Randomisation | “I said well, no I don’t mind and, in a way, the whole point of the trial, as far as I am concerned is that I didn’t know!” (0202)  “But no, I don’t really have in that sense I realise that I could be, it’s randomly selected and I could be in either of the two groups and I’m happy to participate regardless of which group I am in. Ultimately if it helps the research to draw some conclusions, that’s the main purpose” (0318) |
|  | Questionnaires | ‘That you would want so you would say well, I guess I’m in the nearest one to that, you know, do you do it frequently; infrequently; rarely and that kind of thing and I think oh, I’m not sure which box to tick here. I feel I fall often somewhere between one or two of the boxes and so yes, that’s true of many questionnaires.’ (0318)  “It was alright. I am not a great one for filling in paperwork but no problem really.” (0202) |
|  | Receiving the intervention | “Well, no effort from my point of view except I couldn’t stand up. Because when you are doing the balance, because I can’t balance very well, I was going over. So he ended up doing it 2/3 times.” (0116) |
| **Behaviour and support during study procedures** | Foot-care | “Didn’t realise how important it was to really look after the feet.” (0110) |
|  | Participation rationale | “It may be a bit late in life for it to affect me, but the generations that come along afterwards, surely they deserve the better treatment if it transpires that there is a better treatment, so that’s why, purely and simply for future generations!” (0208) |

Additional File 1 (continued) Identified narratives of themes and sub-themes from patient participant interviews

| **Behaviour and support during study procedures** | Family | “My wife has to tell me to take them off when I go out because I forget. She says ‘go change your shoes’.” (0114) |
| --- | --- | --- |
|  | Study support | “When I got there, they made me very welcome. They explained to me what they were going to do and didn’t rush anything and it was very good. She sat me on that couch and explained everything to me about what they were going to do; what they did.” (0205) |
|  | House shoes | “Tend to use them mostly when I’m in the house for any length of time or sort of, when you first get up in the morning and things like that.” (0318) |
| **Impact of study involvement** | Location & logistics | Gives me a day out, really, a ride on the bus and the boat but that’s the only thing! (0208) |
|  | Diabetes impact | “It’s just that I’ve got to be careful about what you eat and what not.” (0202) |
|  | Overall Experience | To be negative about it, I would say there are no negatives as far as I am concerned, that might sound you know good or bad I don’t know. From my perspective, it’s worked 100%.” (0307)  “Perhaps one day you won’t have to use all those wires and that. Do you know what I mean? Perhaps the old computer could do exactly the same job, do you know what I mean because it looked a bit old fashioned. Connecting you up to all those cables like they got now, but it done the job, really good!” (0205)  “I enjoyed it, yes I did enjoy it…… I just say that I found it interesting” (0103) |
|  | Receiving the study results | “Well, purely out of interest because you’ve been involved in something, it would just be interesting to know a) If I’ve got the real one or not and b) Whether the trial proved that there is an area there that can be improved if we do whatever, or there is scope for improvement.” (0208) |
