# Supplementary material for: Insoles to ease plantar pressure in people with diabetes and peripheral neuropathy: a feasibility randomised controlled trial with an embedded qualitative study
Source: Pilot Feasibility Stud. 2023 Feb 3;9:20. doi: 10.1186/s40814-023-01252-y (PMC9896776; doi:10.1186/s40814-023-01252-y)
Supplement: Supplementary file 2 — Additional file 2. Identified narratives of themes and sub-themes from podiatrist interviews. [file 40814_2023_1252_MOESM2_ESM.docx]

*Additional File 2- Identified narratives of themes and sub-themes from podiatrist interviews*

| **Theme** | **Subtheme** | **Podiatrist narratives** |
| --- | --- | --- |
| **Accepting the study methods** | Recruitment to study | “I think we did ok, but I’m still disappointed we didn’t reach our target because I thought that would be easily achievable within the time and I can’t think as to why we didn’t meet that target; I still don’t know. Were people asking? I’m sure they were because I was involved I definitely annoyed them with my emails every week so!” (03P) |
|  | Intervention delivery | “Quick and easy! Yeah, em, I think that sums it up, really. Em, there’s no real theory to how we make them. You go to the algorithm of what type of insole you’re going to put in for that particular patient. But what I come back to is it’s fast, it’s quick and that what we want!” (01P)  “I did find it quite time-consuming and there was a lot of information on the first one! The second wasn’t so bad but on the first initial assessment, I found it was quite a lot.” (02P) |
|  | Technology | “I mean, for a long time, it didn’t work because we had IT issues. Yes, the equipment, sometimes I had a few issues with the Fscan, and then, of course, we couldn’t download… we couldn’t get the sensor reader to work with the computer, there was all that issue.” (02P) |
| **Behaviour and support during study procedures** | Podiatrist networks | “Good to highlight the team. I knew a few of them, but it was good as you can get contact numbers so that you could discuss things between each other it’s a good way of networking in the research team.” (01P) |
|  | Training | “Yes, the training session was good really enjoyed it, and well delivered and I think probably there were lots of questions I didn’t ask that maybe I should have. Um, I think what might have been helpful for me was to do a scenario there after the training had been delivered.” (03P) |
|  | Participant interaction | “From a patient point of view, it’s good for us to use it as a tool to show them what we are doing and make it more understandable. I found all the participants to be willing and able, all very pleased and happy to take part.” (01P) |

*Additional File 2 (continued) Identified narratives of themes and sub-themes from podiatrist interviews*

| **Impact of study involvement** | Overall participation | “…and just being part of the research process as well being able to see things from your perspective as well and understanding about how to avoid pitfalls about setting up a project and I’ve done research myself but not on this sort of large scale, so that’s been really interesting as well.” (02P) |
| --- | --- | --- |
|  | Change in practice | “Yeah, it’s made me stop and think about what we do, you know you’ve just got you, you do things out of habit a lot of the time because that’s how you’ve always done them. And then you use the F Scan and you suddenly think oh, actually, that’s not doing what you expected it to do.” (02P) |
|  | Dissemination | “I would like to discuss the findings with the team. I don’t know if you’d be happy coming down and presenting your findings to the team but I think that would be amazing to show clinicians who have been involved in it.” (03P) |
